# B-Lymphocytes from a Population of Children with Autism Spectrum Disorder and Their Unaffected Siblings Exhibit Hypersensitivity to Thimerosal

**DOI:** 10.1155/2013/801517

**Published:** 2013-06-09

**Authors:** Martyn A. Sharpe, Taylor L. Gist, David S. Baskin

**Affiliations:** Department of Neurosurgery, The Methodist Neurological Institute, 6560 Fannin Street, Scurlock Tower No. 944, Houston, TX 77030, USA

## Abstract

The role of thimerosal containing vaccines in the development of autism spectrum disorder (ASD) has been an area of intense debate, as has the presence of mercury dental amalgams and fish ingestion by pregnant mothers. We studied the effects of thimerosal on cell proliferation and mitochondrial function from B-lymphocytes taken from individuals with autism, their nonautistic twins, and their nontwin siblings. Eleven families were examined and compared to matched controls. B-cells were grown with increasing levels of thimerosal, and various assays (LDH, XTT, DCFH, etc.) were performed to examine the effects on cellular proliferation and mitochondrial function. A subpopulation of eight individuals (4 ASD, 2 twins, and 2 siblings) from four of the families showed thimerosal hypersensitivity, whereas none of the control individuals displayed this response. The thimerosal concentration required to inhibit cell proliferation in these individuals was only 40% of controls. Cells hypersensitive to thimerosal also had higher levels of oxidative stress markers, protein carbonyls, and oxidant generation. This suggests certain individuals with a mild mitochondrial defect may be highly susceptible to mitochondrial specific toxins like the vaccine preservative thimerosal.

## 1. Introduction

Autism spectrum disorder (ASD) is a complex developmental disorder characterized by abnormalities of verbal and nonverbal communication, stereotyped restricted interests, repetitive behavioral patterns, and impairment of socialization. ASD now affects 1 in 88 children in the USA [1, 2]. In Great Britain, the costs of supporting children with ASD amount to be *£*2.7 bil/yr, while for adults these costs amount to *£*25 bil/year [3]. Recent studies have estimated that the lifetime cost to care for an individual with an ASD is $3.2 mil [4]. In the USA individuals with ASD have medical expenditures 4.1–6.2x greater than those without ASD, with median expenditures being almost 9 times greater [5, 6]. ASD is usually diagnosed before 4 years of age and has a 5 : 1 male to female gender bias. Although it is believed that multiple interacting genetic and environmental factors influence individual vulnerability to ASD, none have been reproducibly identified in more than a fraction of cases. In addition to complex gene-environment interactions, the heterogeneous presentation of behavioral symptoms within the spectrum of autistic disorders suggests a variable and multifactorial pathogenesis.

*Mercury.* Mercury is a ubiquitous environmental contaminant, that is, transformed into the volatile neurotoxins methylmercury and ethylmercury. In the United States, more than 8500 water bodies in 45 states and territories are listed as impaired for Hg in water, sediments, and/or fish tissue, including many sites lacking a point source of Hg pollution [7]. In addition to the environmental inorganic/organic mercury assaults many children have been exposed to ethylmercury in the form of thimerosal (called thiomersal in the UK, marketed as Merthiolate in the USA) has been used as a preservative agent for vaccines and toxoids [8]. The relationship between thimerosal and ASD has become a very debated topic over the last decade and some researchers have suspected a causal link [9–12]. Large-scale epidemiological surveys have disputed a causal link between ASD and thimerosal exposure [8, 13–16]. The concentration of mercury in the blood of infants and children receiving vaccines with thimerosal has been reported to be very low and without any effects [11]. Therefore, thimerosal is still recommended as a cheap and stable vaccine preservative in some countries.

*Mitochondria.* Evidence that an underlying mitochondrial encephalopathy is associated with ASD has been produced by a number of studies [17–20], although the connection is not universally accepted [21, 22]. A disturbed bioenergetic metabolism underlying autism has been suggested by the detection of high lactate levels in many ASD patients, indicating a mitochondrial oxidative phosphorylation dysfunction in these children. Reduced levels of respiratory mitochondrial enzymes, ultrastructural mitochondrial abnormalities, and a broad range of mitochondrial DNA mutations suggest a linkage between autism and mitochondrial disorders [2]. In addition, markers indicative of elevated steady state levels of oxidative stress are found in the body fluids of ASD individuals and *in vitro *cell studies [23–27]. Recent media attention has been focused on the case of Hannah Poling, a young girl with mitochondrial encephalopathy and autistic features, whose parents won compensation under the United States National Vaccine Injury Compensation Program [28]. It is clearly important to know if a subpopulation of children were/are hypersensitive to the toxic effects of mercury and its compounds.

*Cell Growth or Cell Death?* ASD is a disease associated with a neurological deficit that may be caused by neurodegeneration during development or by a lack of cell growth during brain development, either *in utero *or post-*utero* [1]. While some studies have suggested that the brains of children with autism are oversized [29], many have demonstrated diminished populations of cells such as the Purkinje cells in the cerebellum [30], defects in white matter [31], and functional connectivity [32]. A large number of stressors have been implicated as causative agents in ASD, but only two, valproate and thalidomide, have been definitely shown to cause ASD in both humans and in animal models of ASD. What is so interesting about these two very different compounds is that valproate [33, 34] and thalidomide [35–37] both inhibit mammalian cell proliferation. We designed our growth study to identify if there was a differential effect of thimerosal on B-cell growth, drawn from the families with an affected child, compared with the general population.

## 2. Materials and Methods

B-cells from ASD individuals, their unaffected fraternal twins, and their unaffected nontwin siblings were obtained from the Autism Genetic Resource Exchange collection (AGRE; Los Angeles, CA, USA). Unaffected sex and age matched external controls were obtained from the Coriell collection (Coriell Cell Repository, Camden, NJ, USA). This design was chosen to allow comparison of the familial ASD genotype with external controls and to also allow comparison between same and different *in utero* environments on the development of ASD. Many potential environmental triggers of ASD have been examined in a toxicological setting. We have elected to monitor cell growth as our metric so we could identify if ethylmercury, in the form of thimerosal, significantly inhibited cell growth in cells drawn from ASD familial genotypes with respect to non-ASD controls.

Cells were grown in 96-well plates where 84 wells were inoculated with 270 *μ*L of 100,000 cells/mL in the presence of 0, 25, 50, 100, 250, 500, and 1000 nM thimerosal. 10 *μ*L of thimerosal was added to each well as an ethanolic solution, some 60 minutes prior to inoculation, and the ethanol was allowed to evaporate. We use one series of 12 wells as internal controls for the assays and these were filled with 270 *μ*L of growth medium. On each plate we grew three ASD, twin, sibling, and Coriell controls at each of the thimerosal concentration. On day 0, 7 plates were inoculated and 6 were placed in an incubator. Each day a plate was removed from the incubator and underwent the following analysis.

*LDH Assay.* 2 × 30 *μ*L of cell suspension was assayed for the levels of lactate dehydrogenase activity in the absence and presence of detergent [38–40]. The final assay mixture was comprised of 110 mM lactate, 3.35 mM NAD^+^, 350 *μ*M resazurin and 2.2 units/mL of diaphorase in 3 mM Tris/30 mM HEPES/10 mM NaCl buffer (pH 7.4), and 0.45% Triton X-100 for total LDH. The resorufin formed was measured over the course of 15 minutes in a plate reader using 530/25 nm ex and 590/35 nm em. The rate of resorufin formation is proportional to the level of LDH. Total cellular LDH was recorded in the presence of detergent, whereas dead/dying cells were recorded in the absence of detergent. The relationship between cell numbers and LDH levels is calculated by measuring LDH and comparing this to the number of cells counted in a cell counter.

*XTT Assay.* 1 × 40 *μ*L of cell suspension was withdrawn to be assayed for mitochondrial function/number using the XTT (2,3-Bis(2-methoxy-4-nitro-5-sulfophenyl)-2H-tetrazolium-5-carboxanilide) assay method [41–43]. This assay consists of adding cell suspension to 40 *μ*L of XTT (1 mg/mL) diluted in media and the XTT is then converted to formazan by the mitochondrial reductase in metabolically intact cells. After 30 minutes of incubation, 40 *μ*L of stop solution (40% SDS in 1 : 1 water : ethanol) was added and the formazan product was measured at 570 minus 650 nm. The signal was quantified using authentic XTT formazan.

*ROS Basal/Antioxidant Levels*. 4 mL of cell suspension was grown for 5 days in 5 mL wells and then washed in 2xPBS. They were assayed for protein and then resuspended on PBS/15 mM glucose and plated into wells at 30 *μ*L of 0.5 mg/mL. Cells from 96-well plates were washed once in PBS and were diluted to *≈*500,000 cells per mL. 2 × 30 *μ*L of cell suspension was assayed for the ability to oxidize 2′,7′-dichlorofluorescein diacetate (DCFH-DA). 30 *μ*L of cell suspension was added to 60 *μ*L of 120 uM DCFH-DA in PDB/15 mM glucose, in the presence and absence of 900 *μ*M hydrogen peroxide. The kinetics of dichlorofluorescein generation is monitored at 428/20 nm ex and 528/20 nm em, for 2 hours and for 15 minutes, respectively. The signal is quantified using authentic dichlorofluorescein.

*Lactate.* 50 *μ*L samples of cell media were acidified using TCA to a final concentration of 400 mM in order to precipitate protein, the plates were then centrifuged, and 40 *μ*L samples were removed and neutralized with 400 mM NaOH. 50 *μ*L aliquots were then removed and added to 50 *μ*L of LDH assay mixture containing 3.1 mM NAD^+^, 130 uM hydrazine sulphate, and LDH 20.8 U/mL, in PBS. The formation of NADH was monitored at 340–380 nm over 40 minutes.

## 3. Results

### 3.1. Representative Growth Curves and the Calculation of LDH-G_50_ and XTT-G_50_

**Figure 1:**
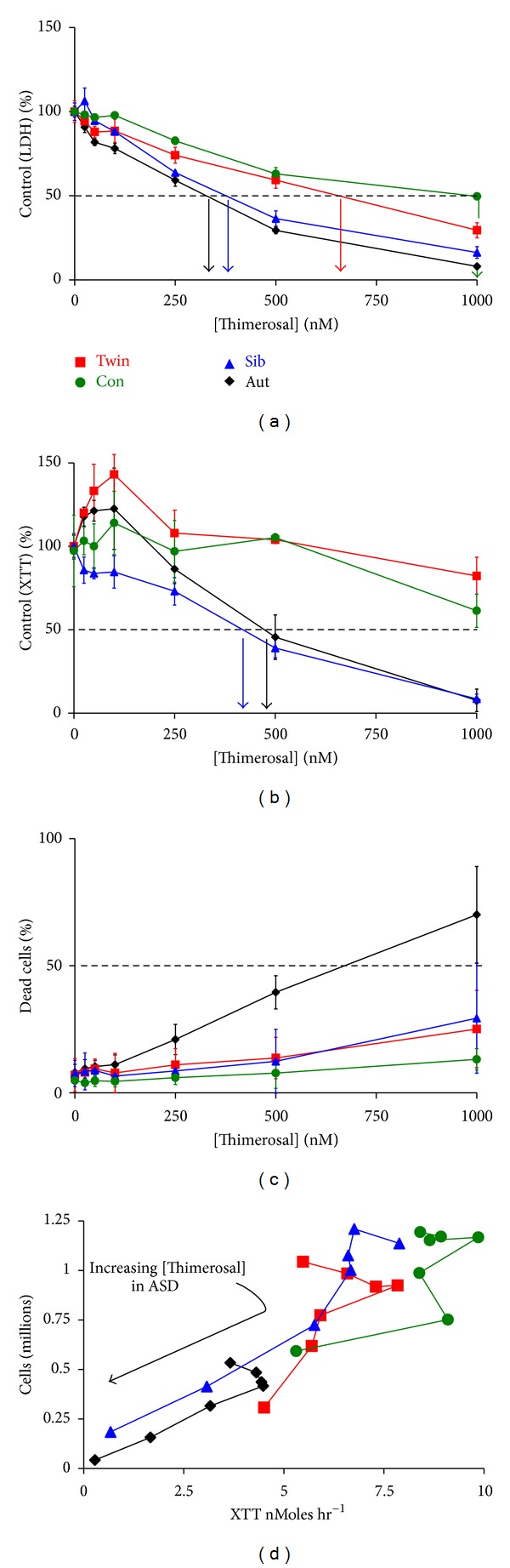
This figure shows the typical responses of a family of cells to thimerosal, measured on day 5 postinoculation. ASD = black, Twin = red, Sibling = blue, Control = green. (a) The percent control LDH values are plotted against increasing thimerosal concentrations for the 4 cell types. Arrows indicate LDH-G_50_ values with ASD = 314 nM, Twin = 648 nM, Sib = 373 nM, and Cont = 1000 nM. (b) The percent control XTT values are plotted against increasing thimerosal concentrations. XTT-G_50_ values are obtained using the mitochondrial XTT assay. (c) The percentage of cellular LDH that is accessible to lactate (i.e., percentage of dead and dying cells) is plotted against thimerosal. (d) Cell number and XTT reduction rates as a function of increasing thimerosal concentrations are shown. The plots obtained are “hockey stick” in shape.

Figure 1 shows a typical dataset from one family, Family B.  Figure 1(a) shows % LDH levels for the ASD, Twin, Sib, and control cells with respect to thimerosal concentration. The arrows indicate the LDH-G_50_ values, ASD = 314 nM (Black), Twin = 648 nM (Red), Sib = 373 nM (Blue), and Cont = 1000 nM (Green); the same color scheme for each cell line is used throughout this paper. In Figure 1(b), the XTT-G_50_ values obtained using the mitochondrial XTT assay are shown. At <250 nM thimerosal one observes an increase in the levels of XTT reduction compared with the untreated cells. This upregulation is a feature of low thimerosal treatment and is present in >70% of the cells examined. It is also obvious from Figure 1(b) that the calculation of the XTT-G_50_ is problematic when it is >1000 nM, and estimates of XTT-G_50_ >1000 nM obtained from semilog plots have to be treated with caution.  Figure 1(c) shows the percentage of cellular LDH, that is, accessible to lactate (i.e., percentage of dead and dying cells). While the death rate correlates well with both of the G_50_s, at neither G_50_ concentration is there an equal number of dead and living cells. Thus, thimerosal is inhibiting cell proliferation as well as causing cell death. The LD_50_ for thimerosal in the ASD cells is 680 nM, more than twice the LDH-G_50_ value of 314 nM and a third larger than the XTT-G_50_. This means that concentrations of thimerosal that do not induce significant cell death can profoundly affect cell proliferation. The cells shown in Figure 1 have a background death rate between 7 and 10%. Increasing this rate to 20% leads to a growth reduction of 60% of the control in the ASD cells, 45% of control in the Twin cells, 25% of control in the Sib cells, and, by projection, to 30% of control in the Coriell control cells. Finally, in Figure 1(d) we show the relationship between cell numbers and XTT reduction rate in the four cells types as a function of thimerosal concentration. These plots exhibit “hockey stick” slopes, where low thimerosal appears to cause the B-cells to restrict their proliferation and use their resources to upregulate their mitochondrial numbers. After this reallocation of resources in response to low levels of thimerosal, there is a steady fall in the cell population which tracks mitochondria function as the concentration of thimerosal is increased.

### 3.2. The Distribution of Thimerosal Sensitivity in the Four Cell Types

**Figure 2:**
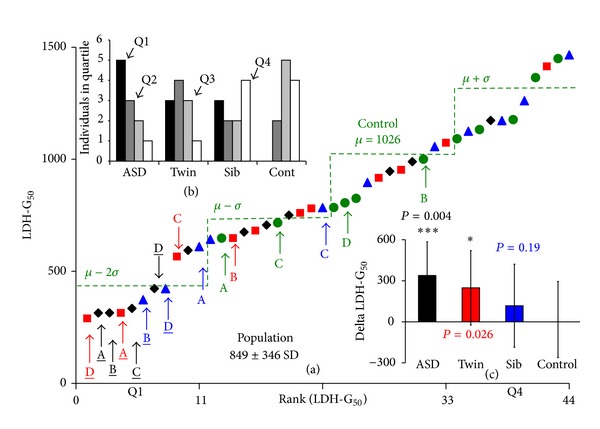
(a) shows the thimerosal concentration that induced a 50% growth inhibition at day 5, measured by the LDH method (LDH-G_50_). In (a) the color-coded cells are ranked in terms of sensitivity to thimerosal. Four family groups are shown whose autistic B-cell LDH-G_50_'s fall greater than 2 standard deviations below the mean: A, B, C, and D. Underlined letters denote those cells believed to have a heightened sensitivity to thimerosal (i.e., those falling outside two standard deviations of the control population). The distribution of the control population is indicated by the green lines showing mean + SD, mean, mean – SD, and mean − 2SD. *Distribution of Cells Types.* In the upper insert, (b) we show the distribution of the four cells types: ASD, unaffected Twin, unaffected Sibling, and external age/sex matched control, in the four quartiles of the ranked distribution. It is noteworthy that the ASD derived cells are more clustered in the left hand side of the distribution and the external controls are distributed to the right hand side. *Test for Systemic Errors.* In the second insert, (c) we show that there is no systemic correlation between low LDH-G_50_'s in cells drawn from families from an ASD background and their respective controls. The graph in (c) shows the average and standard deviation of the difference between the appropriate age/sex matched control and each of the ASD familial LDH-G_50_. The positive values indicate that cells from the affected families are more sensitive than the external controls. The *P* values indicate the results from a one-tailed *t*-test, *n* = 11, with, (*) indicating <0.05, and (***) indicating <0.005.

In Figure 2 the LDH-G_50_s obtained for all 44 cell lines are shown. It is also apparent that there are two populations of cells: those hypersensitive to thimerosal, like the ASD and Sib shown in Figure 1, and a more robust hyposensitive population, like the Twin and Cont of Figure 1. The population distribution of the LDH-G_50_, that is, generated by varying thimerosal concentrations is shown in Figure 2, measured on Day 5 postinoculation. In Figure 2(a) we have ranked the color-coded cells in terms of sensitivity to thimerosal and have highlighted four family groups: A, B, C, and D. Underlined letters denote cell lines believed to have a heightened sensitivity to thimerosal. Figure 2(b) shows the distribution of the four cells types within the four quartiles of ranked distribution. It is quite evident that ASD cells are very much overrepresented on the left hand side of the ranked plot and controls on the right hand side. Also evident from the distribution of the controls for family groups A to D is the possibility of a systemic error to the distribution, indicating that low G_50_s in cells from the hypersensitive ASD families have low scoring external controls. Such an error could be caused, for instance, by differences in different batches of growth media which predispose cells to thimerosal sensitivity or, hypothetically, a difference in the amount of thimerosal added to each growth well. To test for a systematic error, the difference data was plotted (e.g., ASD A-Cont A, Twin A-Cont A) and tested to see if there was a difference in the three populations with respect to the control. Figure 2(c) shows the average and standard deviation of the difference between the appropriate age/sex matched control and each of the ASD familial LDH-G_50_. The positive values indicate that cells from the affected families are more sensitive to thimerosal than the external controls. The *P* values indicate that the whole ASD and Twin populations are significantly different from the controls.

*The Statistics of the LDH-G*_50_ *Distribution.* The Coriell control cells for the other 7 families (i.e., families E–K, those families not having autistic B-cell LDH-G_50_s less than 2 standard deviations below the mean) have a mean LDH-G_50_ of 1026 nM, with a SD of 295 nM, and the 21 hyposensitive cell lines (ASD, Twin, and Sib) of families E to K have a mean G_50_ of 985 nM, with a SD of 259 nM. In contrast, the 12 cell lines (ASD, Twin, and Sib) of families A to D have a statistically different mean LDH-G_50_ of 452 nM and a SD of 167 nM. The distribution of cells is best split into two types (hyper- and hyposensitive) with four ASD individuals (Families A to D), two twins (Families A and D), and two siblings (B and D) being hypersensitive to thimerosal, with all the other cells being the same as the control population (see Figure 2 in Supplementary Material available online at http://dx.doi.org/10.1155/2013/801517 for plotted data). One-tailed *t*-tests to determine if these individuals belong to a more sensitive population, with respect to the Coriell controls, give *P* values of 0.0003, 0.003, and 0.007 for ASD, Twin, and Sib, respectively.

**Figure 3:**
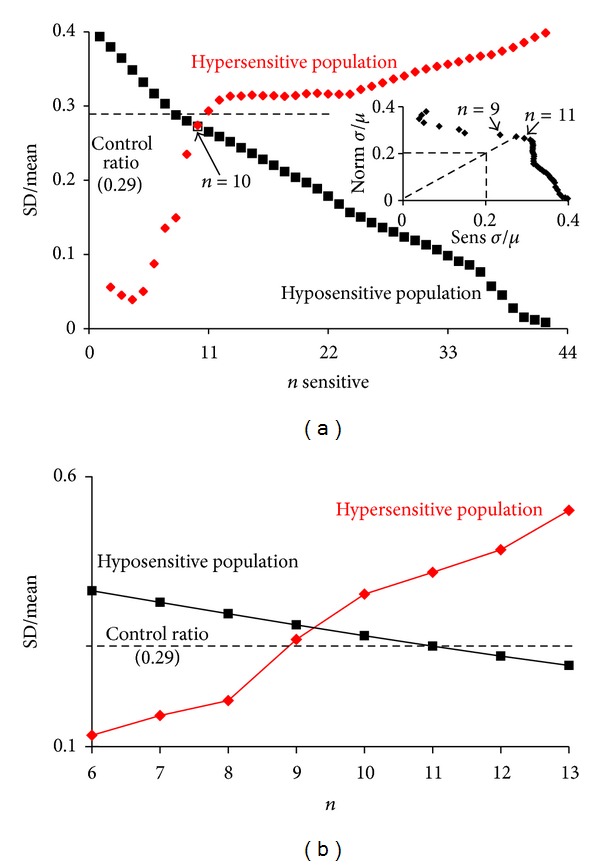
This figure shows two methods to estimate the size of the thimerosal sensitive population, by using a pseudo-Jackknife statistical procedure (a) and by simulation (b). In (a) the ranked data set shown in Figure 2 was treated as a bimodal population. We calculate the mean and standard deviation where the size of the hypersensitive population, *n*, was increased from 0 to 44 and the bulk population fell from 44 to 0. The ratios of the two SDs divided by their means were plotted against *n*. The dashed line is the ratio of the SD/mean of the control population. The insert shows the line-shape generated when the two ratios are plotted against each other. This pseudo-Jackknifing procedure indicates that the size of the hypersensitive population is at least 8 and could be as high as 11. The ranked data were also fitted by simulation to two populations with means of *≈*380 and *≈*100 nM, respectively. The simulations indicated that the two populations had the same population distribution when *n *was 9, (b).

*What Is n?* The population was further tested for the degree of bimodalism and for the size of the sensitive population, *n*, in two ways. Firstly, a pseudo-Jackknife statistical procedure was performed whereby the means and standard deviations of the ranked data were calculated for increasing sizes of hypersensitive population *n*; that is, for a hypersensitive population of *n* = 8, the hyposensitive population is 36. The two standard deviations/means were then plotted against the size of the hypersensitive population (Figure 3(a)). This treatment of the data gives a visual demonstration of the methodology of Holzmann-Vollmer Test for bimodality [44]. In Figure 3(a) it is apparent that as the size of the hypersensitive population is increased its *σ*/*μ* ratio rises and the *σ*/*μ* ratio of the hyposensitive population falls. In the (hyposensitive) Coriell controls the *σ*/*μ* ratio is 0.29 and that value is reached for the hyposensitive population when *n* = 8. At *n* = 10, the two *σ*/*μ* ratios are equal, indicating that at this point the two hypo/hypersensitive populations have the same Gaussian distribution, and the only difference in the populations is the means.

In the insert of Figure 3(a) the same *σ*/*μ* ratio data is shown, but in this case the two ratios are plotted against each other. It is evident that 8 ≤ *n* ≤ 10 as this is the inflection point of the plot. 

It is clear from the two plots shown in Figure 3(a) that there are two populations of cells with differing thimerosal sensitivity. 

We also attempted to define *n* using simulation. We fitted the LDH-G_50_ curve presented in Figure 2 with two normal distributions where the two *σ*/*μ* ratios were allowed to vary between 0.1 and 0.6. We found that by simulation, the best fit occurred when *n* = 9, Figure 3(b). When *n* was increased >9 the *σ*/*μ* ratio of the hypersensitive population became unreasonably large, and at *n* < 9 the *σ*/*μ* ratio of the hypersensitive population became unreasonably small. 

If *n* = 9, then the LDH-G_50_ of the hypersensitive population is 372 nM and has a standard deviation of 87 nM and the hyposensitive population has a mean of 972 nM and a standard deviation of 272 nM. Given the size of the two populations, 9 and 35, and the two distributions we expect that in the first quartile of the ranked data there would be all 9 hypersensitive cell lines and 2 hyposensitive cell lines.

**Figure 4:**
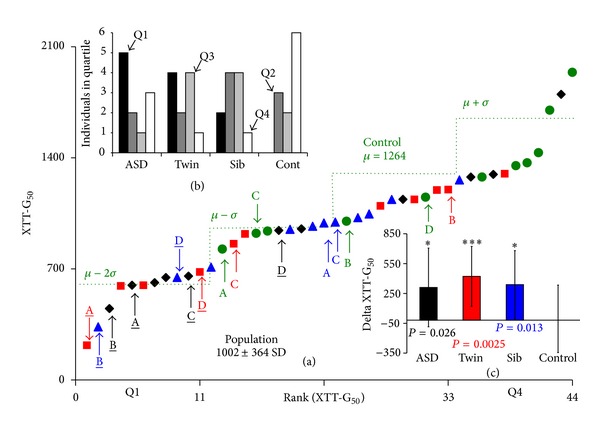
This figure shows the concentration of thimerosal which induced a 50% inhibition of growth at day 5, measured by the XTT method (XTT-G_50_). In (a) we have ranked the color-coded cells in terms of sensitivity to thimerosal and have highlighted four family groups: A, B, C, and D. Underlined letters denote cell lines we believe have a heightened sensitivity to thimerosal. The distribution of the control population is indicated by the green line that indicated mean + SD, mean, mean – SD, and mean − 2SD. *Distribution of Cells Types.* In the upper insert, (b) we show the distribution of the four cells types, ASD, unaffected Twin, unaffected Sibling, and external age/sex matched control, in the four quartiles of the ranked distribution. There is less clustering of the ASD derived cells, but as with the LDH assay, the external controls are distributed to the right hand side of the rankings. *Test for Systemic Errors.* In the second insert, (c) we show that there is no systemic correlation between low XTT-G_50_'s in cells drawn from families from an ASD background and their respective controls. The plot in (c) shows the average and standard deviation of the difference between the appropriate age/sex matched control and each of the ASD familial XTT-G_50_. The positive values indicate that cells from the affected families are more sensitive than the external controls. The *P* values indicate the results from a one-tailed *t*-test, *n* = 11, with (*) indicating <0.05 and (***) indicating <0.005.

*The Statistics of the XTT-G*_50_ *Distribution.* A similar effect of thimerosal on the ability of cellular mitochondrial complexes to reduce XTT was also found. In Figure 4, the XTT-G_50_ obtained for all 44 cell lines is shown with 8 ≤ *n* ≤ 10 cell lines again shown to be hypersensitive to thimerosal. The population distribution of the XTT-G_50_, measured on day 5 postinoculation is shown in Figure 4(a) and again the different cell types are color-coded. Figure 4(b) shows the distribution of the four cell types within the four quartiles of the ranked distribution. ASD cells are again very much over-represented on the left hand side of the plot and controls on the right hand side. Figure 4(c) shows the average and standard deviation of the difference between the appropriate age/sex matched control and each of the ASD familial XTT-G_50_. The positive values indicate that cells from the affected families are more sensitive to thimerosal than the external controls. The *P* values indicate that all three types of cell lines from the ASD families are significantly more sensitive to thimerosal than are the controls drawn from the general population.

*Are Mitochondria the Target in the Hypersensitive Population?* It is quite clear from Figures 1, 3, and 4 that the ability of cells to be able to reduce XTT is linked to their growth. This can be more easily seen in Figure 4 Supplementary Material where the ranked ratio of LDH-G_50_/XTT-G_50_ is shown. The cells identified as hypersensitive to thimerosal have a much greater LDH-G_50_/XTT-G_50_ than the controls and also to other cells drawn from ASD families. There is no difference in the distributions of the *n* = 11 ASD, Twin, and Sibs, when compared to controls. However, if the 8 ASD, Twin, and Sibs we have previously identified as being hypersensitive to thimerosal are compared to the remaining 36 cells, we find that they are statistically significant with a *P* value of 0.024 in a *t*-test. However, the ability of mitochondria to reduce XTT does not directly track either LDH-G_50_ or cell death. We appear to see an upregulation of mitochondrial activity at low thimerosal levels and then a decline in the ability to reduce XTT at higher thimerosal levels.

**Figure 5:**
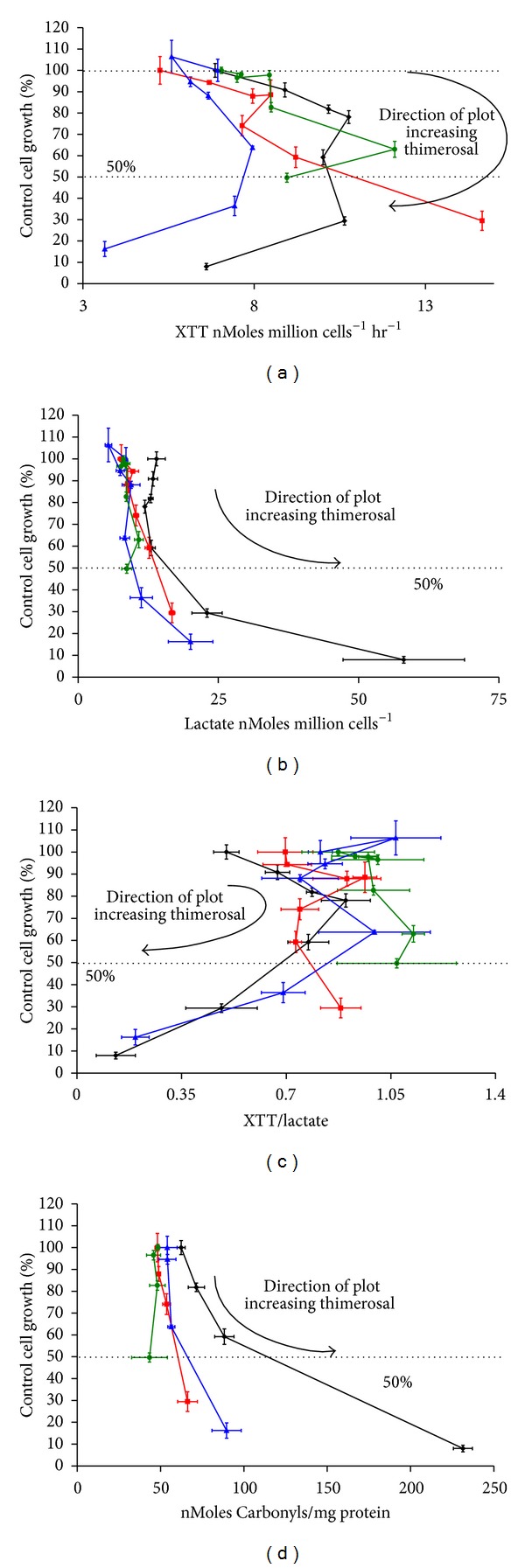
This figure shows data from Family B only, part of the same data set presented in Figure 1. (a) shows the rate of XTT reduction per million cells versus the % control cell growth for increasing thimerosal concentrations. (b) shows the rate of lactate production per million cells versus the % control cell growth for increasing thimerosal concentrations. (c) shows the XTT/lactate ratio versus % control cell growth for increasing thimerosal concentrations. (d) shows the amount of nMoles of carbonyl per mg of protein produced versus the % control cell growth for increasing thimerosal concentrations.

In Figure 5, data that shows mitochondria are the primary target of thimerosal in the hypersensitive populations is presented.  Figure 5(a) shows the rate of XTT reduction per million cells versus the % cell growth for increasing thimerosal concentrations. These data are of Family B and are part of the same data set presented in Figure 1. What is evident is that there is an initial upregulation of mitochondria, followed by a collapse in both cells and mitochondria, in the sensitive cells. The cells can also generate ATP by glycolysis, producing lactate.  Figure 5(b) shows how the levels of lactate per cell are increased in the hypersensitive cells, in response to increasing levels of thimerosal. Moreover, it is evident that the ASD cells have a higher proportion of their energy generation from glycolysis than the other cells. This is best seen by examining the ratio of XTT reduction divided by lactate generation versus cell growth, Figure 5(c). A low XTT/lactate ratio is indicative of a high rate of glycolysis and a low rate of oxidative phosphorylation. Both hypersensitive cells (ASD and Sib) have a crash in their XTT/lactate ratios that correlates with the onset of falling growth, the increase in cell death (Figure 1(c)), and the manifestation of markers of oxidative stress.  Figure 5(d) shows the levels of protein carbonyls, measured using the dinitrophenyl hydrazine method, with respect to thimerosal concentration. Again, both of the hypersensitive cells show an increase in oxidized protein levels, with the ASD cells being severely damaged at LDH-G_50_ levels. What is also very interesting is that the background levels of the two hypersensitive cells were higher than the two hyposensitive cells. The Coriell controls and Twin reported background levels of 48.37 ± 1.2 and 48.1 ± 1.43 nM/mg protein, respectively, whereas the Sib and ASD reported 54.05 ± 5.9 and 62.34 ± 12.39 nM/mg protein, respectively (*n* = 6). This is consistent with either an increase in the rate of reactive oxygen species production, as was reported in ASD cells drawn from AGRE versus controls from Coriell by James et al. [27], or a decrease in the rate of ROS detoxification, as measured by the DCFH-DA method.

Similar measurements were performed in two families, Family B and Family H. The latter family of cells was picked as they were the most representative of the Coriell control population with a LDH-G_50_ of 1014 ± 348 and a XTT-G_50_ of 1102 ± 340, giving SD/mean ratios of 0.34 and 0.31.  Table 1 shows the rate of DCFH oxidation for cells drawn from Families B and H. Twelve independent measurements were taken for each family and averaged to yield the means (*μ*) and standard deviations (*σ*) shown. A statistically significant difference in the steady state oxidant levels is seen only in the ASD cells drawn from Family B, *P* = 0.000121. There is also a rise in the background of the (hypersensitive) Sib, but it is not statistically significant. This is rather similar to, but less than, the increase in the oxidant levels observed in the two cell types by James et al. [27].

That the basal level of oxidant levels in these ASD cells is 50% higher than in the controls does not inform as to whether the cells are generating more oxidants, if they have worse antioxidant defenses, or if they have a combination of both. To dissect out the intrinsic difference in the rate at which oxidants are generated by a particular cell type and the state of their antioxidant defenses due to thimerosal, the rate of DCFH oxidation in the presence of a hydrogen peroxide load was measured. Cells were grown in either the absence or presence of 250 nM thimerosal, washed, and then loaded with DCFH and presented with an oxidative insult in the form of a 100 *μ*M H_2_O_2_ addition (see Table 2). In this experiment, the level of DCFH generation is independent of the low levels of oxidants generated by the cells and is only dependent on the activity of the various antioxidant defenses. A cell with robust antioxidants will generate less fluorescent oxidized DCFH than will cells with an antioxidant defect. Family H, which appears hyposensitive to thimerosal, shows a 20-fold increase in the rate of DCFH oxidation in the presence of external hydrogen peroxide and this rate is not affected by the presence of 250 nM thimerosal in their growth media; *n* = 6. The three cell lines of the ASD family all show poorer antioxidant defenses than the control and all the members of Family H. The ASD cells of Family B show a 23-fold increase in oxidation rate after addition of 100 *μ*M H_2_O_2_ but start from a much higher level. Moreover, there is a clear statistically significant difference in the rate of DCFH oxidation in the ASD cells grown in the presence and absence of 250 nM thimerosal (*P* = 0.01). We believe this difference is evidence of an inherent defect of antioxidant defense in the cells derived from this particular ASD individual. The changes induced in the other three cell types of Family B were not statistically significant.

## 4. Discussion

In this study we have examined the action of low levels, ≤1000 nM, of thimerosal on immortalized B-cells taken from ASD subjects, their fraternal twins, a sibling, and from an age/sex matched control. We have examined such 11 family groups and identified 4 families, with at least 8 and as many as 10 members of these families, who have a hypersensitivity to thimerosal. The concentration of thimerosal required to inhibit cell proliferation in these individuals is only 40% that required in hyposensitive controls. We have shown that in these hypersensitive cells mitochondria are the target organelle conferring thimerosal sensitivity. Cells that can maintain mitochondrial energy production are robust, showing little effects on growth, on lactate generation, or on cell death levels. Cells that lose mitochondrial function show increased levels of lactate generation and higher death rates.

Recently James et al. [27] examined B-cells derived from ASD individuals drawn from the AGRE collection and compared them to unaffected controls from the Coriell collection. In their study they examined the effect of three hours incubation with thimerosal, 0.156 to 2.5 *μ*M, on endogenous oxidative stress in B-cells. We have independently conducted a similarly designed study but examined the effects that thimerosal, 0.05 to 1 *μ*M, has on cell growth over the course of six days. Cell numbers, measured using the lactate dehydrogenase method [40] modified to read the fluorescence of resorufin generated by NADH/Diaphorase [42], and mitochondrial function, measured by the XTT reduction rate [41, 43], were examined in eleven family groups of B-cell lines from AGRE consisting of an ASD individual, an unaffected fraternal twin (who shared the same *in utero* environment), an unaffected sibling, and an age/sex/ethnically matched control from a separate population (Coriell). We have also measured the media lactate levels [45] and the levels of protein carbonyls to assess levels of anaerobic respiration. We report that we find that the sensitivity towards thimerosal amongst these cells is bimodal, with *≈*18% of the cells (all AGRE) displaying more than 2.5x the sensitivity to thimerosal as the rest of the population.

*Thimerosal. Methylmercury, Ethylmercury, and Inorganic Mercury in Baby's Blood and Brains.* We used concentrations of thimerosal which reflect the *in vivo* concentrations in infants and newborns following vaccinations. Stajich and coworkers [10] examined the levels of blood mercury prior to and following a single dose of a thimerosal (12.5 *μ*g) containing vaccine and found that in term infants the concentration rose to an average of 11 nM, but that in preterm infants this was 36.7 ± 24.4 nM SD, indicating that 15% of the infants may have a blood concentration of >60 nM. The estimated blood half-life of mercury after administration of thimerosal in babies is between 5 and 7 days [46]; however, mouse [47] and primate [48] studies indicate that both organic and inorganic mercury levels in brain have a much longer residency (>3 weeks). The concentration of organic/inorganic mercury in brain is typically higher than in blood as organomercury partitions into the lipid rich environment. The partition coefficient into the brain of thimerosal derived organic/inorganic mercury in young primates is 5-6. Thus, blood organic/inorganic mercury concentrations reflect one-sixth of the brain levels [48]. The concentration range that reflects brain organic/inorganic mercury postthimerosal vaccination is in the order of 100 and 500 nM, drawing on the epidemiological data of Ball, Stajich & Burbacher, and coworkers [12, 48, 49].

We find evidence for oxidative stress being a contributing mechanism in hypersensitivity. Cells which are hypersensitive to thimerosal also have higher levels of oxidative stress markers, protein carbonyls, and higher levels of oxidant generation. These same cells also showed compromise of their antioxidant defenses after being grown in the presence of low levels, 250 nM, thimerosal. Our findings are consistent with the only other thimerosal/B-cell study that found mitochondria were thimerosal targets and that antioxidant defenses, especially those linked to glutathione, were intrinsically compromised in ASD cells and were further eroded by exposure to thimerosal [27]. This completely independent study also shows that when an ASD B-cell population is hypersensitive to thimerosal, the twins/siblings have a 50% chance of being the same. The hypersensitivity of twins and siblings was directly dependent on having a familial ASD who was hypersensitive. It implies that there is a genetic component to thimerosal hypersensitivity and this hypersensitivity is only found in a third of the ASD sufferers. This supports a multi-insult model of ASD causation where many individuals have the genetic background that makes them vulnerable to a particular type of insult at a particular time in their brain development; that is, one-third of ASD sufferers couldhave a genetic predisposition to mitochondrial/antioxidant insults [9, 18], one-third a genetic predisposition to *in utero* testosterone exposure [50], and the final third a genetic predisposition to toxoplasmosis [51]. 

ASD is a disorder caused by a problem in brain development. If the B-cells from the families in the AGRE collection are at all representative of the neurons in the brains of the cell donors, we can say that a third of them have a sensitivity to thimerosal that would restrict cell proliferation at levels that were/are typically found after vaccination [11, 12, 16, 46–48].

Moreover, we find that hypersensitive populations have poorer antioxidant defenses, elevated markers of oxidative stress, and high lactate levels. These findings are consistent with a metabolic fingerprint typically found in 20% of ASD individuals, plasma hyperlactacidemia [52].

Although we have established that a third of our ASD subjects have a heightened sensitivity to thimerosal, and this sensitivity is shared by one-third of their twins/siblings, this study does not address etiology. What we suggest is that although standard toxicology studies of thimerosal indicate a LDH-G_50_ of 1000 nM with a SD of 300 nm, a minority of subjects from a discrete subpopulation have a LDH-G_50_ of <350 nM with a SD of <100 nm. In our recently published work, we have shown that the mitochondria of normal human astrocytes accumulate the ethylmercury lipophilic cation and that after this primary insult cell death occurs [53]. Here we show that a subpopulation of four individuals with autism, along with some of their siblings, have B-cells exhibiting hypersensitivity toward thimerosal that can be attributed to their mitochondrial phenotype. Thus, certain individuals with a mild mitochondrial defect may be highly susceptible to mitochondrial specific toxins like the vaccine preservative thimerosal. 

## Supplementary Material

 Figure 2A Supp shows the color-coded ASD-ranked LDH-G_50_ in each of the eleven AGRE family groups, A-K, and Coriell external controls. The boxes represent three different means; +/- SD. In families A-D, the 12 individual cells lines have an average LDH-G_50_ of 452 nM, with a SD of 167. The 21 cell lines of families E-K have an average LDH-G_50_ of 985 nM, with a SD of 259. The eleven Coriell external controls cells have an average LDH-G_50_ of 1017 nM, with a SD of 308. Families A-D are significantly different from controls while the other families are not; with *p* values of one-tailed t-test's being 0.0000068 and 0.1697 respectively.
Figure 2B Supp shows the same data plotted with respect to cell type divided into cells believed to be thimerosal sensitive (red) and normal (black). The *p* values on the plot indicate the results from a one-tailed t-test with respect to the whole (i.e. n=11), Coriell population. The datasets strongly suggest that at least 8 cell lines, 4 ASD, 2 Twins and 2 Siblings, are members of a discrete population in which the LDH-G_50_ is 348 nM with a SD of 52.
Figure 4 Supp shows the effect of thimerosal on the ratio of LDH-G_50_/XTT-G_50_, derived from the data shown in Figure 2 and Figure 3. In Figure 4A Supp we have ranked using our color-coded cell labeling system and have again underlined cell lines we believe highly sensitive to thimerosal. In the upper insert, Figure 4B Supp, we show the distribution of the four cells types in the four quartiles of the ranked distribution. ASD derived cells are more clustered in the right-hand side of the distribution and the external controls are distributed quite evenly. In the second insert, Figure 4C Supp, we show the average and standard deviation of the difference between the control and familial cell ratios. The ASD cells are not statistically different from the controls, or both sets of siblings. However, the sensitive cells that we have identified are characterized by having a large LDH-G_50_/XTT-G_50_ ratio and their distribution is clearly skewed to the right.

## Figures and Tables

**Table 1:** The rate of DCFH oxidation for cells drawn from Families B and H. Twelve independent measurements were taken for each family and averaged to yield the means (*µ*) and standard deviations (*σ*) shown. The ASD cell type was the only cell type that differed significantly between the two families (*P* = 0.000121).

Cell Type	DCFH Oxidation nMole mg^−1^ hr^−1^					
	Fam B *µ*	Fam H *µ*	Fam B *σ*	Fam H *σ*	% Coriell B	% Coriell H
**ASD**	3.56***	**2.52**	**0.237**	**0.121**	**148**	**87**
Twin	2.44	2.14	0.170	0.076	101	74
Sib	2.65	2.52	0.088	0.093	110	87
Cont	2.42	2.90	0.107	0.080	100	100

**Table 2:** The DCFH oxidation (nMole mg^−1^ hr^−1^ **)** of cell families B and H after addition of 100 *µ*M H_2_O_2_ and 250 nM thimerosal. ASD B-cells from family B showed a significant difference (*P* = 0.01) of DCFH oxidation when exposed to thimerosal. None of the other cells lines in B or H showed any statistical difference. This indicates an inherent defect of antioxidant defense in the ASD population of cells.

Cell ID	DCFH oxidation with 100 *µ*M H_2_O_2_	DCFH oxidation with 100 *µ*M H_2_O_2_ and 250 nM Thimerosal	Difference
ASD-H	49.85 ± 4.34	48.25 ± 2.02	−1.61
Twin-H	49.32 ± 6.19	49.31 ± 2.42	−0.01
Sib-H	48.35 ± 6.7	43.34 ± 2.35	−5.01
Cont-H	47.15 ± 4.8	41.48 ± 5.46	−5.67

**ASD-B**	**83.5 ± 13.76**	**93.86 **±** 13.76**	10.396**
Twin-B	61.32 ± 6.19	60.41 ± 10.69	2.963
Sib-B	62.55 ± 6.7	58.22 ± 10.81	3.816
Cont-B	46.93± 4.8	42.03 ± 17.6	4.990

**Indicates statistically significant difference.
